# Experiences before and after nasogastric and gastrostomy tube insertion with emphasis on mealtimes: a case study of an adolescent with cerebral palsy

**DOI:** 10.1080/17482631.2021.1942415

**Published:** 2021-06-24

**Authors:** Ulrika Mårtensson, Mats Cederlund, Margaretha Jenholt Nolbris, Karin Mellgren, Helle Wijk, Stefan Nilsson

**Affiliations:** a Institute of Health and Care Sciences, Sahlgrenska Academy, University of Gothenburg; University of Gothenburg Centre for Person-Centred Care (GPCC), Gothenburg, Sweden; b Institute of Neuroscience and Physiology, Sahlgrenska Academy, University of Gothenburg, Gothenburg, Sweden; c Institute of Health and Care Sciences, Sahlgrenska Academy, University of Gothenburg, SE-405 30, Gothenburg, Sweden and Queen Silvia Children’s Hospital, Sahlgrenska University Hospital, Gothenburg, Sweden; d Department of Paediatrics, Institute for Clinical Sciences, Sahlgrenska Academy, University of Gothenburg, Gothenburg, Sweden; e Department of Quality Strategies, Region Västra Götaland, Sahlgrenska University Hospital, Gothenburg, Sweden; f Department of Architecture and Civil Engineering, Chalmers Technology University/Centre for Health Care Architecture, Gothenburg, Sweden

**Keywords:** Adolescents, cerebral palsy, gastrostomy tube, mealtimes, nutrition

## Abstract

**Purpose**: Adolescents with cerebral palsy may need a feeding tube due to feeding challenges, since nutritional intake and mealtimes may be negatively affected. The purpose of the study was to describe and better understand how one adolescent with cerebral palsy and her parents experienced mealtimes before and after a nasogastric and gastrostomy tube insertion and how the use of these feeding tubes was experienced in daily life.

**Methods**: Individual interviews were performed with one adolescent and each of her parents. In total, six interviews were conducted on two separate occasions. The qualitative approach known as Interpretive Description was used during the analysis.

**Results**: Four thematic patterns were identified within the data: (i) struggling with nutritional intake, (ii) the paradox of using an aid, (iii) being different, and (iv) challenges of public mealtimes.

**Conclusions**: The results showed that four themes influenced daily mealtimes in adolescents with cerebral palsy and a gastrostomy tube. Nutritional intake and mealtimes may be difficult, which is why using a gastrostomy tube can be a relief. However, the gastrostomy tube can also pose a challenge and a paradox. Time of change and acceptance seems necessary in order to meet these challenges.

## Introduction

This case is about an adolescent with cerebral palsy (CP), referred to by the pseudonym Lisa. Deteriorated scoliosis was the origin of her suffering, which, in combination with the scoliosis treatment caused side effects such as behavioural changes regarding nutritional intake and mealtimes. Thus, the goal of pain management was to achieve acceptable pain relief. However, the pain and its side effects could not be adequately relieved. Consequently, Lisa stopped feeding orally and eventually relied on enteral nutrition (EN) via a feeding tube.

In CP, inadequate nutritional intake is a serious problem (Kim et al., 2018), and adolescents with CP often experience feeding challenges (Graham et al., 2019; Remijn et al., 2019; Sewell et al., 2014). Several factors may affect the adolescent’s need for energy, e.g., reduced ability to movement, which may complicate the nutritional assessment (Quitadamo et al., 2016). Factors such as dysphagia (Graham et al., 2019; Quitadamo et al., 2016; Remijn et al., 2019; Sewell et al., 2014) give rise to unsafe oral intake, which affects the adolescent’s overall ability to eat (Adams & Roy, 2014; Quitadamo et al., 2016). This is to say that individual aspects associated with the function and structures of the body as well as the environment may affect the health of adolescents with CP (World Health Organization Geneva, 2002). Further, pain is a common experience (Alriksson-Schmidt & Hagglund, 2016) that may influence the daily lives, for instance, intake of food and fluids, to a high degree (Jayanath et al., 2016). Also, medications such as opioids (Matson et al., 2019) can cause side effects, such as nausea and vomiting (Efune et al., 2018), which can contribute to a loss of appetite. Therefore, adolescents with CP are at risk of poor growth and malnutrition (Leonard et al., 2020; Quitadamo et al., 2016; Sewell et al., 2014), which can negatively affect both their overall health and well-being (Leonard et al., 2020).

Difficulties with food consumption can have an adverse effect on adolescents with CP, causing unpleasant emotions for them during mealtimes (Remijn et al., 2019). Mealtimes can be time consuming (Quitadamo et al., 2016; Sewell et al., 2014) and associated with a feeling of distress (Remijn et al., 2019; Sewell et al., 2014). If the adolescent with CP is not monitored at mealtimes, this can become a further obstacle that may cause the adolescent to become overwhelmed due to the required adjustments and aids (Remijn et al., 2019). Due to these environmental challenges, making changes to food—for instance, consistencies—or avoiding food altogether—which may be one approach that the adolescent with CP use to cope with the situation (Remijn et al., 2019).

Since health is associated with an experience of well-being in different areas and levels (World Health Organization (WHO), 2021), the paediatric care team has a responsibility to give adolescents with CP an opportunity to reach this state (World Health Organization (WHO), 2021, UNICEF, 2021), which is why EN (Adams & Roy, 2014; Quitadamo et al., 2016) via a feeding tube may become necessary (Heuschkel et al., 2015; Quitadamo et al., 2016). A feeding tube can have positive effects in adolescents with CP, since their condition—physical issues (Mahant et al., 2018; Nelson et al., 2015; Quitadamo et al., 2016), nutrition status (Caselli et al., 2017; Quitadamo et al., 2016)—may improve as well as their overall health and well-being (Nelson et al., 2015; Quitadamo et al., 2016). A feeding tube may also decrease stress (Quitadamo et al., 2016), serving as a helpful alternative (Russell et al., 2018) that may reduce parental concerns regarding food (Mahant et al., 2018).

In adolescents that need EN during a limited period, a nasogastric tube (NG-tube) may be an option, while a gastrostomy tube (G-tube) can be advantageous from a long-term perspective (Quitadamo et al., 2016; Scarpato et al., 2017). The insertion of an NG-tube does not require a surgical procedure; the feeding tube is easy to remove but can result in bodily discomfort associated with the nose and sinuses (Quitadamo et al., 2016; Scarpato et al., 2017). Despite the invasive procedure (Quitadamo et al., 2016; Scarpato et al., 2017), it can be advantageous to receive a G-tube for adolescents with CP due to the added comfort (Quitadamo et al., 2016; Scarpato et al., 2017) and neat appearance (Quitadamo et al., 2016). However, deciding whether an adolescent with CP requires a feeding tube is difficult for the parents (Adams & Roy, 2014; L Edwards & Leafman, 2019; Mahant et al., 2018), and involving the whole family in the decision is necessary (Graham et al., 2019; Lee & Corden, 2019; Mahant et al., 2018).

The empirically grounded modified version of the Five Aspect Meal Model (M-FAMM) (Mårtensson et al., 2021) was developed from the Five Aspect Meal Model (FAMM) (Gustafsson, 2004; Gustafsson et al., 2006; J S A Edwards & Gustafsson, 2008). The FAMM, which incoroprates—the room, the meeting, the product, the management control system and the atmosphere, focused on improving mealtimes and the mealtime experience at restaurants (Gustafsson, 2004; Gustafsson et al., 2006; J S A Edwards & Gustafsson, 2008). The FAMM (Gustafsson, 2004; Gustafsson et al., 2006; J S A Edwards & Gustafsson, 2008), has been tested and validated in children with cancer, i.e., a short-term use of a G-tube in a hospital environment, with the conclusion that an adaption of the model was needed (Mårtensson et al., 2021). Consequently, the M-FAMM, developed and adapted specifically for children with a G-tube, includes two further aspects, bodily discomfort and time of change and acceptance (Mårtensson et al., 2021), see Table I. The M-FAMM (Mårtensson et al., 2021) has been evaluated using criteria developed by Risjord (Risjord, 2019) and seems to be an appropriate meal model to use in children with a G-tube. Thus, the M-FAMM (Mårtensson et al., 2021) constitutes the theoretical framework employed in this study in order to further test and validate the model in a new context, i.e., in an adolescent with CP in a home environment with a long-term need for a G-tube.

**Table I.** Examples of the aspects included in the M-FAMM (Mårtensson et al., 2021)

Aspects of the M-FAMM	Examples
*The room*	The environment in the room, e.g., sounds, lights, colours, furnitures, textiles
*The meeting*	E.g., the child’s participation, communication and interaction during mealtimes
*The product*	E.g., the taste and visual impression of the food
*The management control system*	The ability to choose food and influence the timing of meals
*The atmosphere*	E.g., smells as well as feelings and well-being during mealtimes
*Bodily discomfort*	E.g., the child’s experience of discomfort and pain stemming from the G-tube
*Time of change and acceptance*	Experiences before, during, and after the G-tube insertion Transition process

Until today, few studies have been conducted regarding how adolescents with CP experience the use of an NG- or G-tube. Further, it is still unclear how adolescents with CP experience mealtimes before and after an NG- or G-tube insertion and their impact on mealtimes in daily life. By interviewing an adolescent with CP and her parents, this important area can be highlighted and better understood. In addition, the M-FAMM (Mårtensson et al., 2021) can be examined further in order to clarify if this meal model can also be used in the context of children with disabilities in paediatric care. Consequently, the purpose of the present study was to describe and better understand how one adolescent with CP and her parents experienced mealtimes before and after an NG- or G-tube insertion and how the use of these feeding tubes is experienced in daily life.

## Methods

### Study design

Using a case study design makes it possible for the researchers to highlight and understand a specific phenomenon (Ebneyamini & Moghadam, 2018; Merriam, 1998; Yazan, 2015)—in this case mealtime experiences. In this study, it also provided the opportunity to capture the situation as a whole and thereby gain more detailed knowledge (Ebneyamini & Moghadam, 2018; Yazan, 2015) about an adolescent’s journey from a straightforward to complex nutritional situation. The researchers’ goal was—in combination with the study design—to carry out in-depth individual interviews (Carter et al., 2014).

The M-FAMM (Mårtensson et al., 2021) can be seen as a way to interpret others’ lived experiences. Since the M-FAMM (Mårtensson et al., 2021) constituted the theoretical framework for the study, the aspects of the model also acted as a lens for the first author (UM), as it provided her with an insight into the family’s mealtimes during the research process, and thus influenced the interpretation of the interviews. Due to this, the researchers chose to use semi-structured interviews (Polit & Beck, 2017), which, in this study, were quite similar to in-depth interviews, which means that they alternated between being controlled and organized to being more open and free in their questions (Carter et al., 2014). The first author (UM), who performed all the interviews, encouraged the participants to discuss other matters in the area of feeding tubes and mealtimes which they felt were important, thereby giving more depth to the data (Carter et al., 2014).

Researchers can use in-depth individual interviews and focus groups in order to perform data source triangulation (Carter et al., 2014). In-depth interviews are useful when the researcher’s focus is to deeply understand and explore the participant’s experiences (Carter et al., 2014). In this case, the research focused on Lisa’s perspective, but the researchers chose to add her parents’ external observations as a proxy perspective as well. Semi-structured interviews with Lisa’s parents were used with the aim of performing data source triangulation (Carter et al., 2014). Triangulation across the data sources (Carter et al., 2014; Ebneyamini & Moghadam, 2018; Merriam, 1998; Polit & Beck, 2017; Yazan, 2015), in this case comparing Lisa’s, her mother’s and her father’s experiences regarding mealtimes, increased both the depth and trustworthiness of the study (Carter et al., 2014; Ebneyamini & Moghadam, 2018; Merriam, 1998; Yazan, 2015).

### Case description

There are several systems for classifying and assessing physical and psychological functions in CP (Eliasson et al., 2006; Hidecker et al., 2011; Palisano et al., 1997; Sellers et al., 2014). The Gross Motor Function Classification System (GMFCS) (Palisano et al., 1997), the Manual Ability Classification System (MACS) (Eliasson et al., 2006) and the Communication Function Classification System (CFCS) (Hidecker et al., 2011) evaluate an individual’s possibility to move (Palisano et al., 1997), use the hands when managing regular tasks (Eliasson et al., 2006) and communicate (Hidecker et al., 2011). However, the Eating and Drinking Ability Classification System (EDACS) is a way to classify an individual’s capability to eat and drink (Sellers et al., 2014) and seems to be an effective method for detecting dysphagia and aiming to identify typical complications, such as malnutrition, in adolescents with CP (Ron et al., 2020). The GMFCS (Palisano et al., 1997), the MACS (Eliasson et al., 2006), the CFCS (Hidecker et al., 2011) and the EDACS (Sellers et al., 2014) are divided into five different levels, where the first level has a minimal negative impact while level five is the most severe and includes strong limitations for the individual (Eliasson et al., 2006; Hidecker et al., 2011; Palisano et al., 1997; Sellers et al., 2014).

This case involved a 14-year-old adolescent with CP and both of her parents. The adolescent, Lisa, had eaten orally without difficulties her whole life, but at the age of 13, issues revolving around her nutritional intake arose, which negatively affected mealtimes. Lisa’s nutritional intake worsened due to her scoliosis, which caused her pain. Lisa had no cognitive dysfunction and communicated verbally without needing to use alternative communication methods. Lisa exhibited symptoms of level I of CFCS; because the paediatric care team did not find it relevant to perform an assessment, thus she attended a regular state school. Lisa was categorized as GMFCS level V and MACS level II by a physiotherapist and the paediatric care team. Lisa’s ability to move was limited, but she needed to use a travel service every day to get to school. As a result, she required a personal assistant.

Physicians prescribed drugs such as opioids in order to manage the pain Lisa experienced due to scoliosis. The use of these drugs probably contributed to the side effects Lisa described, for instance, nausea, vomiting and lack of appetite. By the time the study was conducted, these symptoms constituted a significant problem in Lisa’s daily life, which led to problematic mealtimes where all attention was on Lisa’s nutritional intake. In Lisa’s case, the symptoms resulted in escalating feeding difficulties, including prolonged mealtimes, marginal nutritional intake, physical weakness and acute weight loss. Lisa went from eating healthily to eating minimal amounts. As a result, Lisa received a feeding tube (an NG-tube) in December 2017. In January 2018, the NG-tube was changed to a G-tube, and in May 2018, the G-tube was changed to a low profile tube. The EDACS assessment was not performed by the paediatric care team before or after each feeding tube change, since it was clear that Lisa constituted level I of the classification system. Lisa’s need for a feeding tube was not related to any physical obstacles. Therefore, Lisa could receive nutrition and perform mealtimes safely.

### Data collection

Purposive sampling was used at a habilitation centre. The inclusion criteria for the study was an adolescent with CP who had experiences of different types of feeding tubes, i.e., an NG- and G-tube. This case study is part of a larger project examining children who, for medical reasons, have received a G-tube. In addition, the children must be five years or older or have a cognitive developmental level of five years or older.

The adolescent’s perception was essential, since the adolescent’s own perspective needs to be explained (Nilsson et al., 2015; Söderbäck et al., 2011). The parents’ interviews involved an adolescent perspective and were important in order to achieve a deeper and more varied perspective regarding mealtimes (Hemmingsson et al., 2017; Longo et al., 2017; Söderbäck et al., 2011). The participants chose their home as the interview location, and all interviews were carried out in Swedish. Each participant was individually interviewed twice during a follow-up period of four months, with a total of six interviews each lasting between 30 and 43 minutes. The interviews were carried out independently at two different time points: once in May 2018 and once in September 2018. The interview guide, which was based on the M-FAMM (Mårtensson et al., 2021), was similar to the one given to Lisa and her parents (see Table II).

**Table II.** Examples of how the M-FAMM (Mårtensson et al., 2021) influenced the interviews

Aspects represented in the M-FAMM	Examples of questions in the interview guide/the interviews
*The room*	Tell me about your/your child’s experience regarding the environment in the room during mealtimes.
*The meeting*	Tell me about your/your child’s experience regarding participation, communication and interaction during mealtimes.
*The product*	Tell me about your/your child’s experiences regarding the visual impression of the food at mealtimes. Tell me about your/your child’s experience regarding your/your child’s taste of the food.
*The management control system*	Tell me about your/your child’s experiences regarding your/your child’s ability to choose the food and influence the timing of meals.
*The atmosphere*	Tell me about your/your child’s experience regarding smells as well as feelings and well-being during mealtimes.
*Bodily discomfort*	Tell me about your/your child’s experience of discomfort and pain stemming from the G-tube.
*Time of change and acceptance*	Tell me about your/your child’s experience before, during and after the G-tube insertion.

### Preunderstanding

The first (UM), third (MJN), and last author (SN) have many years’ experience working as paediatric nurses. Additionally, the first (UM) and second author (MC) also have many years’ experience working at a paediatric habilitation centre. The second (MC) and fourth author (KM) have experience in paediatrics, and the fifth author (HW) has experience in caring science.

### Data analysis

The first author (UM) conducted, recorded, and transcribed all the interviews. Since the purpose of this study was to interpret and describe an adolescent’s everyday life, an Interpretive Description (ID) qualitative approach was adopted, as outlined by Thorne (Thorne, 2016; Thorne et al., 2004). In ID, the researchers’ preunderstanding of the study topic is viewed as important and useful, bringing further dimensions to the analysis process (Thorne et al., 2004). In this study, using ID in the analysis allowed the authors to apply a theoretical framework (Thorne, 2016; Thorne et al., 2004), in this case the M-FAMM (Mårtensson et al., 2021). In this way, the researchers were able to understand one adolescent´s experiences, her complexities and the context in a more varied way (Thorne, 2016).

**Figure 1.**
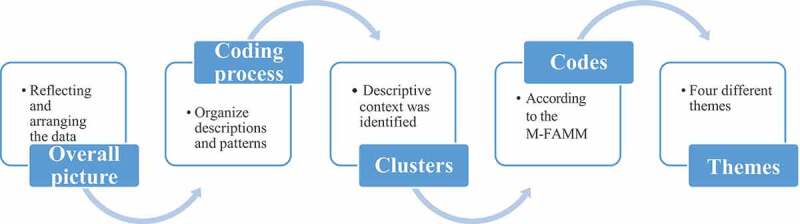
Overview of the analysis process

The analysis consisted of arranging the data and obtaining an overall picture of the case which is essential to the ID process (Thorne, 2016). The next step in the analysis process aimed to organize descriptions and patterns from the collected data (Thorne, 2016; Thorne et al., 2004) and was performed manually. The data was analysed separately by the first and last author (UM, SN), and during regular meetings, the analyses were synthesized and compared in order to find similarities and dissimilarities across the data sources (Carter et al., 2014). In this inductive process a descriptive context was identified that could be grouped into clusters and later coded according to the aspects of the M-FAMM (Mårtensson et al., 2021) (see Figure 1).

**Figure 2.**
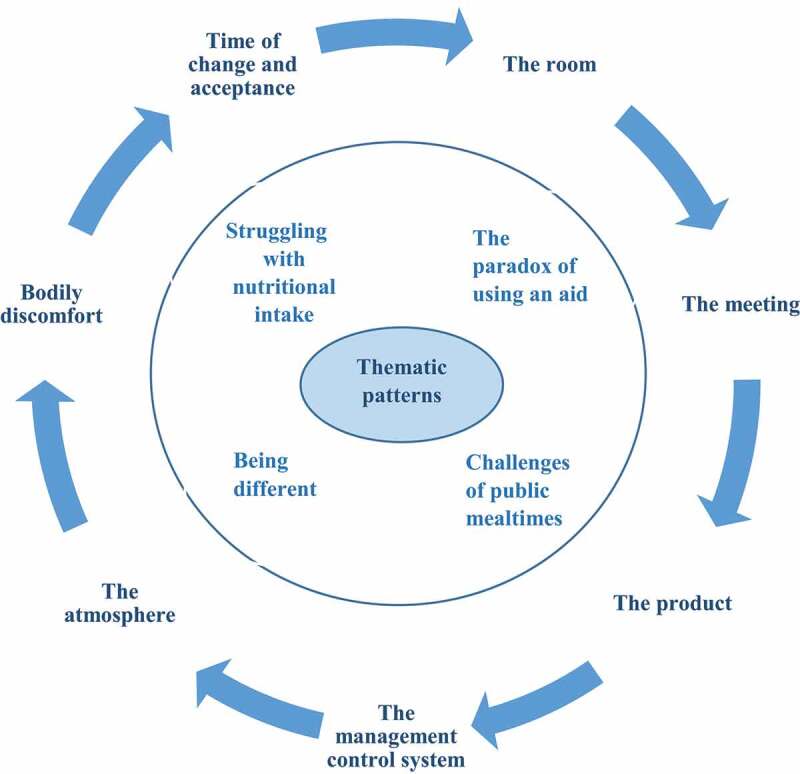
Illustration of thematic patterns in relation to the M-FAMM (Mårtensson et al., 2021)

Data patterns were grouped into themes. In this way, each participant’s experiences could be described and represented, which in turn resulted in meaningful themes being identified by the first author (UM) and checked by the last author (SN). Finally, four different thematic patterns were identified across the data (see Figure 2), and these were processed, discussed and decided upon by all the authors.

To ensure that descriptive data was interpreted in an appropriate manner by the researchers, the first author (UM) returned to the participants during the analysis process and carried out member checks. This enabled the participants to validate, review and verify the collected data which strengthened the validity of the study (Carter et al., 2014; Polit & Beck, 2017; Yazan, 2015); the Standard for Reporting Qualitative Research (SRQR) (Equator network. Standards for reporting qualitative research (SRQR), 2019) was followed. In addition, certain principles (e.g., current trustworthiness from Merriam’s methodology for case studies) (Yazan, 2015) were used as a guide during the research process in order to ensure the quality of the study. The generalizability of the data was also strengthened by obtaining a clear description of the context and characteristics of the participants.

### Ethical considerations

This study was approved by the Regional Ethics Review Board (937–17 approved 13/12/2017 and 2019–05671). The first author (UM) informed the participants both verbally and in writing about the study and obtained their verbal and written consent to participate. Participation in the study was voluntary, and the participants understood that they could withdraw their participation at any time without providing a reason. Adolescents can be vulnerable, especially adolescents affected by the type of diagnosis included in the study. Therefore, the researchers ensured that they maintained a high degree of respect and consideration throughout the research process. In the study, all information was treated confidentially, no unauthorized persons had access to the data, and steps were taken to ensure that the participants were anonymous. All data was stored in a locked, fireproof filling cabinet.

## Results

After examining the individual interviews, four thematic patterns were identified from the data: (i) struggling with nutritional intake, (ii) the paradox of using an aid, (iii) being different, and (iv) challenges of public mealtimes. The results provide insight into the mealtime process and Lisa’s experiences over time before and after the tube insertions. In each theme, the results are presented according to Lisa’s experiences, i.e., before and/or after the insertion of the feeding tubes, ending four months after with the low profile tube insertion.

### Struggling with nutritional intake

Struggling with nutritional intake refers to the period before the NG- or G-tube insertions, where Lisa experienced constant pain and the challenges she faces around mealtimes.

Lisa’s parents unanimously asserted that Lisa’s pain and side effects gradually worsened, severely affecting her appetite, although it took time for them to grasp the real seriousness of the situation: “*Yes, I had probably not realized that it was so bad with the food right then”*(Lisa´s father). Both parents described how Lisa, who had eaten orally with a good appetite all her life, progressively began to lack interest in eating: “ … *Increasingly more and more morphine was given, which made the appetite disappear”* (Lisa´s mother). Bodily discomfort was described as an aspect that may have negatively affected Lisa’s nutritional intake. In this case, serious consequences such as nausea, lack of appetite and weight loss as well as a decline in Lisa’s nutritional intake caused a spiral of negative reactions at mealtimes, and overall deterioration was apparent.

Mealtimes, which had normally been a source of joy for the family, had now been replaced with negative emotions such as stress, pressure, and frustration. More specifically, they had become challenging for Lisa since mealtimes were now focused on ensuring that she was obtaining an adequate nutritional intake. Consequently, Lisa’s desire and motivation to eat completely disappeared. The negative atmosphere during mealtimes affected Lisa remarkably. Her feeding problems steadily escalated, which led to difficult mealtimes for both Lisa and her parents: “ … *It was very stressful at the beginning to make sure that she would get nourishment, therefore one was completely exhausted* … ” (Lisa’s mother). Lisa confirmed that mealtimes during this time were more demanding than enjoyable, which contributed to her feelings of distress: “ … *It consumed more energy than I actually managed to take in”* (Lisa).

Despite all the attempts made to increase her nutritional intake, including taking dietary supplements, it became clear that Lisa was losing weight: “ … *the weight curve went downward … it didn’t work … ”* (Lisa). Lisa’s father expressed worry over the situation: “ *… she lost a lot of weight, as she had difficulty eating and taking in food through the mouth …* ” (Lisa´s father). Insufficient nutritional intake combined with weight loss over time contributed to Lisa’s serious physical situation. Finally, Lisa’s mother felt that, even though her daughter had tried to manage her feeding issues, it was not working anymore. Therefore, she considered a feeding tube as an option for Lisa in order to manage and improve her situation. Lisa’s parents tried to get support from her paediatric care team, asserting Lisa’s need for a feeding tube, since both her health and well-being had been negatively affected. However, they needed to contact the team repeatedly to reiterate the increased need for nutritional support. After a long-term struggle, a decision was made that Lisa needed a feeding tube in order to recover. Lisa was set to have a G-tube but received an NG-tube in the interim.

### The paradox of using an aid

The theme, the paradox of using an aid, came from the period after Lisa had received a G-tube and refers to the tube as being both a solution and an obstacle for the family. In this interpretation, the paradox describes the positives and the ambiguities of living with a G-tube.

The whole family viewed the G-tube as a positive change: “ … *Lisa thought it was wonderful too I think”* (Lisa’s father). The G-tube became a solution to Lisa’s feeding problems, since she no longer dealt with an oral eating requirement. Reduced stress and pressure at mealtimes resulted in less difficulties surrounding Lisa’s nutritional intake. Her parents agreed that the G-tube presented a solution that gave them a sense of control. However, according to Lisa’s parents, the most important advantage of the G-tube was the sense of security it provided. They expressed a sense of relief at having the ability to ensure Lisa’s needs regarding nutrition and medication were met: *“ … what a relief … you have at least the opportunity to give [food] even on a difficult day … or to add the medication … There is a sense of freedom to know … ”* (Lisa’s mother). The G-tube also gave Lisa a renewed sense of control, giving her a new sense of independence and freedom. Mealtimes improved and became relaxed again: *“ … The pressure is not the same … now I have a choice”* (Lisa). The change in atmosphere also had a positive impact on the mealtime experience at home. Overall, the G-tube insertion resulted in Lisa’s improved health and well-being, as increased nutritional intake gave Lisa energy, leading to a more active and functional daily life.

However, despite the G-tube insertion, bodily discomfort—such as nausea and vomiting—remained which proceeded to negatively affect Lisa’s nutritional intake. Due to the nausea, Lisa often felt sick in the mornings making it difficult for her to eat at all. The G-tube had facilitated her food consumption, but because her nausea remained, the bodily discomfort negatively impacted Lisa’s well-being and daily life. It affected her so much that her eating habits needed to be changed: *“ … I feel so nauseous in the morning that I cannot eat the food at home and then I have to eat it at school …* ” (Lisa). Furthermore, it was troublesome for Lisa to consume the required amount of food each day and then retain it: “ … *we had big problems getting the whole amount to go down, and for it to stay down*” (Lisa’s mother).

The G-tube insertion resulted in a continuation of non-oral feeding, which led to frustration for Lisa’s parents. They indicated that the long-term difficulties with food that Lisa faced had been traumatic for her, outlining that she now viewed mealtimes as an obligation and associated food with pressure and stress. Lisa’s parents stated that they often tried to encourage Lisa to eat orally at mealtimes. However, they allowed her to choose her food and the time that she wanted to eat. This was in an attempt to help her feel more involved and motivated at mealtimes. They also tried to promote the meals by presenting the food in an appealing way, since the visual impression of food is important to the mealtime experience, but it did not help. However, Lisa’s parents found that their attempts to make Lisa feel more involved were unsuccessful: “ … *Lisa gets the opportunity to try if she wants. We always ask her if she wants something but … 99 times out of 100 she does not want anything”* (Lisa’s father).

Consequently, Lisa began to skip mealtimes with her family. This came as a relief for Lisa as she experienced this withdrawal as something positive—a quiet moment for recovery on her own. In contrast, her parents saw this as a sign that Lisa was disappearing, isolating herself from them and signified that she was becoming more distant. Therefore, Lisa’s parents tried to physically adapt mealtimes by providing her with another room or space in order to avoid this alienation. Lisa would sit in front of the TV and her meals would be placed there. Consequently, Lisa’s withdrawal resulted in a change in routines and new mealtime habits for the whole family. The physical distance at mealtimes resulted in less social interaction and communication within the family: *“ … We don’t have meals together that often … we all have such different eating habits … ”* (Lisa). Therefore, whilst Lisa’s insufficient nutritional intake was solved with the G-tube, new challenges arose, as neither food nor socialization formed a component of Lisa’s mealtimes anymore. Time for change and acceptance was essential for the family to adjust to the new situation after the G-tube insertion.

### Being different

The theme of being different refers to the family’s different experiences with the two feeding tubes, i.e., the NG- and G-tube.

Lisa had a visible disability all her life, and she was used to receiving reactions and comments about it, which generated a sense of being different. The NG-tube became another visible sign and exacerbated the feeling of being different, causing Lisa to resent negative feelings towards her illness. Physical appearance is important, especially for adolescents, which makes it hard for Lisa to manage the new situation. Her parents described feelings and concerns regarding how strangers would respond to Lisa: “*The fact that everyone would look at Lisa in school … ”* (Lisa’s father). The change in appearance became hard to manage, and Lisa, with support from her parents, struggled to handle the situation. As an adolescent, Lisa wanted to look like everybody else, and she therefore tried to hide the NG-tube: “*Lisa thought it was, it was hard … did not want it to be visible. We struggled to make it as invisible as possible … ”* (Lisa’s mother).

The two feeding tubes given to Lisa—the NG- and G-tube—were experienced very differently by Lisa and her parents. Since the NG-tube became a traumatic experience, it negatively affected Lisa’s well-being. The G-tube insertion thus gave the family a sense of relief since it is an aid that is not visible unlike the NG-tube. However, Lisa still felt different; even though the G-tube was less visible, it still represented that she was different from everyone else, “ … *since the button is on the stomach … it may not be ideal … to pull up my sweater in the middle of the hallway”* (Lisa). The G-tube also resulted in questions from people outside the family, especially at mealtimes, which Lisa found hard to contend with: *“ … I don’t want people to see either … because I don’t want any questions about it”* (Lisa).

Contrary to Lisa’s father, Lisa’s mother also described a sense of feeling different (as a mother). Specifically, she described how tube feeding affected her emotionally and that at times her role was no longer that of a mother, but instead felt more like that of a worker. However, her experience of feeling different decreased over time, and both Lisa and her mother became used to it. Acceptance and an adaptation was necessary to meet the challenges—e.g., unexpected reactions and feelings—as well as to recover a sense of well-being for the whole family after the G-tube insertion.

### Challenges of public mealtimes

The theme of challenges of public mealtimes refers to the period before and after the NG- and G-tube insertion. The theme emphasizes how the environment affected Lisa’s mealtimes.

According to Lisa, public mealtimes were a struggle which resulted in her avoiding places like restaurants, major events and the school cafeteria, i.e., places with many people. The most common public mealtime that had a major effect on Lisa’s daily life was the school cafeteria. The prevailing school mealtime environment, which featured significant amounts of noise, resulted in Lisa having severe difficulties in coping with her mealtimes in the cafeteria: *“ … It is so boisterous, messy and noisy and people are shouting and there is such little space … I can’t deal with it”* (Lisa). Her parents agreed with Lisa that the mealtime environment at school required a lot of Lisa’s strength and energy in order to handle it: *“ … she does not want to go into the school cafeteria at all … it is messy … and loud and there are many people … it is a stressful situation …* ” (Lisa’s mother). The cafeteria and its atmosphere were aspects that negatively influenced Lisa’s mealtime experiences.

Before Lisa received enteral nutrition, she was required to eat at the school cafeteria. When the G-tube insertion was administered, new possibilities arose, including withdrawal from the cafeteria, presenting a solution and new ways for Lisa to cope. The G-tube had made it possible for Lisa to both ensure her nutritional intake at school and, at the same time, avoid an uncomfortable environment. Withdrawal from the school cafeteria was a solution Lisa chose; she saw it as a beneficial option to eat with more privacy, which in turn enhanced her ability to recover and improved her overall well-being at school.

Instead of using all her time and energy to focus on eating in the cafeteria, Lisa now had the energy to socialize and interact with her schoolmates during breaks. Despite Lisa’s own choice not to participate in mealtimes at school, she was regularly encouraged to join her schoolmates to eat in the cafeteria. The school staff did not appear to feel comfortable with Lisa’s choice: *“ … I think the assistant is asking Lisa but she gives her a definitive ‘no’ each time …* ” (Lisa´s father). Thus, time of change and acceptance also appeared to be an important aspect for those outside the family with whom Lisa came into contact with.

## Discussion

This unique case study emphasizes the adolescent’s experience and perspective of mealtimes in conjunction with the use of feeding tubes—an NG- or G-tube—which are valuable and of great importance for paediatric care. Using the M-FAMM (Mårtensson et al., 2021) made it possible for the researchers to describe and better understand the adolescent’s experience of using different feeding tubes as well as how mealtimes could be experienced before and after an NG- or G-tube insertion from a more holistic perspective. The findings have been categorized into four representative thematic patterns: struggling with nutritional intake, the paradox of using an aid, being different, and challenges of public mealtimes. This study demonstrated that nutritional intake and mealtimes may be difficult for an adolescent with CP, since they may be associated with pressure, stress, and frustration. These findings are in line with earlier research that has emphasized mealtimes as being challenging and associated with negative emotions for adolescents with CP (Remijn et al., 2019). The new knowledge line in actually have listened to the adolescent herself regarding experiences of living with an NG- and G-tube.

In this case, Lisa gradually lost her appetite due to pain and side effects, which resulted in an unsustainable situation. The findings highlight the need for greater awareness from the paediatric care team of the effect of bodily discomfort, particularly its effect on nutritional intake and mealtime experience; therefore, the M-FAMM (Mårtensson et al., 2021) could be a useful tool. The pain meant that Lisa experienced a number of complications, such as nausea and vomiting, which affected her nutritional intake and mealtimes. It is well-known that pain negatively impacts daily activities—such as nutritional intake—in adolescents with CP (Jayanath et al., 2016). At the same time, drugs such as opioids may cause bodily discomfort, for instance, nausea and vomiting (Efune et al., 2018). Bodily discomfort is one aspect of the M-FAMM that has been consistently affecting mealtimes negatively (Mårtensson et al., 2021). Insufficient food consumption can be serious for an adolescent with CP (Kim et al., 2018) as it can lead to malnutrition and other negative effects (Leonard et al., 2020). Therefore, the paediatric care team of the adolescent must identify and screen those most at risk (Leonard et al., 2020). One way to do this can be by using the M-FAMM, since it is established that its aspects can contribute to an increased knowledge regarding mealtimes in relation to a G-tube (Mårtensson et al., 2021). Therefore, an awareness and understanding of mealtimes may be advantageous in the care of children and adolescents with a G-tube (Backman & Karlsson, 2021; L Edwards & Leafman, 2019; Mahant et al., 2018), since knowledge regarding the G-tube and its expected consequences is necessary for the family (L Edwards & Leafman, 2019; Mahant et al., 2018).

The outcome of this study showed that the G-tube insertion entailed a physical improvement even if complications such as nausea and vomiting remained. However, bodily discomfort still constituted a part of Lisa’s daily life and thus had a great impact on her overall health and well-being. On the other hand, a G-tube does not seem to worsen vomiting or other gastro-oesophageal reflux (GER) associated symptoms (Kakade et al., 2015). Nevertheless, research has shown that those who receive food soon after a G-tube insertion suffer from nausea and vomiting to a higher degree compared to those who receive delayed tube feeding (Peck et al., 2020). Therefore, this study highlighted some advantages of using the M-FAMM (Mårtensson et al., 2021) such as identifying and managing different aspects, e.g., bodily discomfort, which are important for the mealtime experience when the adolescent lives with a G-tube.

A G-tube even though may be challenging for an adolescent with CP, it could lead to improvements in physical health (Mahant et al., 2018; Nelson et al., 2015; Quitadamo et al., 2016). Receiving a G-tube can be considered a paradox for an adolescent with CP, which is why it is important to identify potential changes after the insertion. Through the lens of the M-FAMM, several important aspects that affect mealtimes after a G-tube insertion have been identified; one such aspect is time of change and acceptance (Mårtensson et al., 2021).

The family in this study experienced a sense of relief when the G-tube was finally inserted, since it resulted in less pressure at mealtimes, which is in line with earlier research (Backman et al., 2021; Mårtensson et al., 2021). It has previously been confirmed that a G-tube insertion can result in an alteration in the family’s daily life (Russell et al., 2018). The G-tube gave the parents a sense of security, but also provided the adolescent with greater independence and autonomy. In addition, it enabled the adolescent to regain some control over her mealtimes. All these aspects contributed to a relaxed mealtime for the family. In order to reach optimal mealtimes after a G-tube insertion, commitment from a paediatric care team has been emphasized (Backman et al., 2021; Backman & Karlsson, 2021; Russell et al., 2018). Nevertheless, the G-tube also required the family to change, giving rise to new mealtime habits. The social coexistence of the family altered, since the adolescent chose to withdraw from mealtimes. Due to their needs, a G-tube insertion can involve modifications at mealtimes, which may affect the family (Russell et al., 2018).

The feeling of being different was intensified for the adolescent in this study after the NG-tube insertion. This feeling remained despite the change to the G-tube. Lisa’s external appearance became different, contributing to a sense of being more conspicuous than before. It has previously been highlighted that a feeding tube can be perceived as an obvious sign of an adolescent’s difficulties, garnering potentially problematic reactions from other people (Nelson et al., 2015). Lisa tried to manage this situation by hiding her tube, which is why she described being relieved when changing to the G-tube.

The transition from an NG-tube to a G-tube can be a positive experience for an adolescent with CP, contributing to beneficial changes in health, well-being, and external appearance (Quitadamo et al., 2016). A G-tube can involve both emotional and practical challenges (Nelson et al., 2015; Russell et al., 2018); therefore, it is crucial for the paediatric care team to give extensive assistance to and provide the adolescent with CP, as well as the parents, with necessary tools to deal with mealtimes and the new situation (Russell et al., 2018). Thus, the M-FAMM could be one suitable model to use since the aspect time of change and acceptance for instance, seems to be crucial in managing the needs of an adolescent after a G-tube insertion (Mårtensson et al., 2021).

In this study, Lisa avoided public mealtimes such as the school cafeteria. Therefore, the environment and atmosphere are aspects that need to be taken into consideration in order to get a holistic view of the mealtimes (Mårtensson et al., 2021). Environmental aspects, such as space and range of food during mealtimes, influence, and are essential, in encouraging adolescents with CP to eat and drink (Remijn et al., 2019). The school environment implies mealtimes with loud noises and a lot of people, which had a profound effect on Lisa. This finding is in line with earlier research that emphasizes that adolescents with CP can have concerns regarding their mealtime environment (Remijn et al., 2019). The family can also perceive challenges with the environment during mealtimes (Russell et al., 2018). Thus, the M-FAMM (Mårtensson et al., 2021) can be a necessary tool to support an adolescent’s well-being in unfamiliar settings.

### Limitations

A limitation of this study is the methodological criticism that is frequently aimed at case studies in general. This includes, for example, criticism of the lack of relevance of a single case due to poor generalizability. However, the purpose of this study is not to create generalizable knowledge but to lead to an in-depth understanding of the studied phenomenon. The findings can help in understanding adolescents’ experiences regarding mealtimes in daily life before and after an NG- or G-tube insertion. Another limitation of this case study is that all of its data came from the family, with only one adolescent with CP and her parents interviewed. No one outside the family, such as health care professionals, assistants or school staff, was interviewed. It is clear that the opinions of one single adolescent cannot accurately reflect those held by every individual with CP, as each individual has his or her own unique lived experience, which affects the generalizability of case studies in general. However, such studies do reveal aspects of individuals’ everyday lives, thereby providing guidance regarding how, for example, mealtime experiences might be perceived when an adolescent with CP lives with an NG- or G-tube. Further, the use of the M-FAMM (Mårtensson et al., 2021) as a theoretical framework may be a methodological issue. Using the M-FAMM (Mårtensson et al., 2021) and its predetermined aspects as a base in the interview guide may have had an impact on the answers. More open questions regarding bodily sensations and mealtimes in general could have resulted in altered types of answers.

### Clinical implications

This unique case study provides a further understanding of mealtimes before and after the insertion of an NG- or G-tube. The results give valuable insight regarding a specific adolescent’s lived experience, which can contribute to a deeper understanding of the area. The findings of the study indicate that insufficient nutritional intake combined with weight loss over time may lead to severe physical conditions for an adolescent with CP. As a result, the paediatric care team needs to be aware of the effect of bodily discomfort in these adolescents, particularly on nutritional intake and mealtime experiences.

By using this in-depth knowledge, nursing students as well as paediatric care teams may receive specific information and education regarding the challenges adolescents with CP may experience regarding mealtimes and feeding tubes. This knowledge may contribute to being better prepared to care for adolescents. The knowledge could also be used to educate parents about the challenges an adolescent can experience regarding mealtimes and the use of feeding tubes. By using the M-FAMM (Mårtensson et al., 2021) as a care guide for adolescents with CP, mealtimes can be charted and adapted to their specific needs. Therefore, the development of new guidelines is crucial to change the practical nutritional care for these adolescents. Further, focusing on a greater number of adolescents with CP being offered a G-tube, or at least receiving it sooner, may be necessary.

### Further research

Further studies involving a larger number of adolescents are needed to get a broader perspective of mealtime experiences with a feeding tube. In addition, it is important to underscore the difference between the mealtime experiences of children with CP in different age groups as well as those of children/adolescents with a long-term use of feeding tubes. This in order to identify similarities and differences in their experiences in order to customize the care and treatment to the specific child/adolescent. It is also important to further investigate how innovations can be developed, e.g., new guidelines and a platform of education, with the purpose of optimizing care, treatment and support for these adolescents.

## Conclusions

This study, which is done from an adolescent’s perspective, emphasizes the adolescent’s own experience of using a feeding tube and its impact on daily mealtimes before and after the insertion. The results showed that four themes influenced the adolescent’s daily life: struggling with nutritional intake, the paradox of using an aid, being different, and challenges of public mealtimes. Nutritional intake and mealtimes may be difficult for an adolescent with CP, and a G-tube can be a solution. However, the G-tube can also pose a challenge and a paradox, why time of change and acceptance seems to be necessary in order to meet challenges that may occur.
